# Prediction error, ketamine and psychosis: An updated model

**DOI:** 10.1177/0269881116650087

**Published:** 2016-05-25

**Authors:** Philip R Corlett, Garry D Honey, Paul C Fletcher

**Affiliations:** 1 Department of Psychiatry, Yale University, New Haven, CT, USA; 2 Roche Switzerland, Basel, Switzerland; 3 Department of Psychiatry, University of Cambridge, Cambridge, UK; 4 Cambridgeshire and Peterborough NHS Foundation Trust, Cambridge, UK

**Keywords:** Drug model, ketamine, psychosis, schizophrenia, delusions, hallucinations, computational psychiatry

## Abstract

In 2007, we proposed an explanation of delusion formation as aberrant prediction error-driven associative learning. Further, we argued that the NMDA receptor antagonist ketamine provided a good model for this process. Subsequently, we validated the model in patients with psychosis, relating aberrant prediction error signals to delusion severity. During the ensuing period, we have developed these ideas, drawing on the simple principle that brains build a model of the world and refine it by minimising prediction errors, as well as using it to guide perceptual inferences. While previously we focused on the prediction error signal per se, an updated view takes into account its precision, as well as the precision of prior expectations. With this expanded perspective, we see several possible routes to psychotic symptoms – which may explain the heterogeneity of psychotic illness, as well as the fact that other drugs, with different pharmacological actions, can produce psychotomimetic effects. In this article, we review the basic principles of this model and highlight specific ways in which prediction errors can be perturbed, in particular considering the reliability and uncertainty of predictions. The expanded model explains hallucinations as perturbations of the uncertainty mediated balance between expectation and prediction error. Here, expectations dominate and create perceptions by suppressing or ignoring actual inputs. Negative symptoms may arise due to poor reliability of predictions in service of action. By mapping from biology to belief and perception, the account proffers new explanations of psychosis. However, challenges remain. We attempt to address some of these concerns and suggest future directions, incorporating other symptoms into the model, building towards better understanding of psychosis.

## Introduction

In 2007, in this journal, we outlined a theory of delusion formation expressed in terms of associative learning theory (Corlett et al., 2007a). It was not the first theory of delusions expressed in this framework (Gray et al., 1991; Miller, 1976), but it did implicate a specific psychological process (prediction error [PE]) and its neurochemical underpinnings in the genesis of delusions. We are very honoured to have been invited to revisit this article, and would like to take this opportunity to discuss the origins of the ideas, the key features of the theory, the evidence that has emerged to support and challenge it, and, importantly, the ways in which it has evolved in the context of a cognitive neuroscience field that has advanced rapidly and helped to shape its development.

We begin with a consideration of what attracted us to the central ideas outline in the original article and what precisely we were hypothesising by invoking PE to account for how delusional thinking might emerge.

## Delusions and PE: The key ideas

We, like many, feel the need for a perspective on the key features of psychosis – delusions and hallucinations – that links the perceptual and inferential aspects of these experiences with the underlying biological mechanisms. Without such a linking mechanism, biological explanations of mental illness will remain incomplete. Computational cognitive neuroscience offers the potential to unite multiple levels of explanation through deployment of computational models that can be plausibly related to activity of brain systems, the instantiation of cognitive processes and to high-level behaviour and experience (Corlett and Fletcher, 2014). Our early attempt to do this drew on a number of areas in the existing literature. In particular, we noted that, going back to Bleuler’s (1908) earliest formulations (Miller, 1976), the delusions characteristic of schizophrenia occur against a background of strange and sometimes bizarre associations and experiences. This theme – linking delusional thinking to associative learning (Hartley, 1749/1976) – was developed more formally (Dickinson, 2001) in the context of the theoretically and empirically rich field of animal reinforcement learning, itself given formal foundations by advances in machine learning (Sutton and Barto, 1998) and artificial intelligence (Widrow and Hoff, 1960). Both of these fields conceptualised a fundamental role of the brain as identifying and updating statistical regularities (or associations), in effect to build an internal model of the world (Conant and Ashby, 1970).

Ensuing and increasingly complex and sophisticated perspectives on this idea have envisaged that this challenge is met through an iterative process of predicting and updating (Adams et al., 2013; Corlett et al., 2009a; Fletcher and Frith, 2009). Briefly, the brain uses prior knowledge (prior experience of associative relationships) to predict what its next input will be, and any mismatch between the prediction and what actually ensues is signalled as a PE. This is sometimes referred to as predictive coding or predictive processing (Clark, 2013). PE is a key drive to new learning in so far as it indicates incorrect predictions and hence a model that may need to be updated.

This simple formulation provided the framework that supported our initial consideration of PE in psychosis. Moreover, it offered us an operationally defined and quantifiable parameter that could readily be applied in analyses of neuroimaging data in humans (Corlett et al., 2004; Corlett and Fletcher, 2012; Corlett et al., 2007b; Fletcher et al., 2001; Murray et al., 2008; Turner et al., 2004). If PE signalling is altered, arising inappropriately or with an anomalous degree of strength or precision, then there would ensue, we argued, a compelling sense that one’s existing model of the world was wrong, that something had changed. Perhaps there would be an enhanced tendency to see spurious associations between stimuli and events that, in reality, were not related. Perhaps too, there would arise a sense of a world that had changed, become more sinister and laden with meaning. And, having established a new set of associations, then these would form the framework dictating how attention might be diverted towards ensuing stimuli and shaping the inferences that arose. Put simply, an aberrant PE signal would lead to new learning, and new learning would engender new expectations that would themselves govern how the individual sampled and interpreted the world. In effect, this could lead to a developing change in which emerging beliefs created the evidence that supported them.

So it may be, we suggested, that delusions begin to emerge. Importantly, this explanatory framework, though far from comprehensive, offered the possibility of linking symptoms to brain processes because associative learning processes, including PE, had all been extensively studied in terms of their underlying neurobiology (Fletcher et al., 2001; Lavin et al., 2005; Schultz and Dickinson, 2000; Turner et al., 2004). Our attempts to establish this link focused on dopaminergic and glutamatergic systems and their interactions, given the evidence that these are critical to PE signalling (Lavin et al., 2005; Schultz and Dickinson, 2000). Since ketamine, an influential and compelling model of early psychosis (Krystal et al., 1994; Pomarol-Clotet et al., 2006), impacts both transmitter systems (Kegeles et al., 2000), this framework enabled a comprehensive account of the drug’s psychotomimetic effects.

This early model, as we review below, has proven useful in contributing to subsequent ideas and research in our groups and beyond. Moreover, it has garnered support from a number of experimental approaches. But, like all models, it was necessarily a simplification. In particular, it focused primarily on the formation of simple associations between stimuli or between cause and effect, and it drew out some basic ideas of how a perturbed PE signal might disrupt association formation as well as perception and attention. But it is important to move beyond deterministic first-order associations: the statistical regularities of the world exist at many levels and interact in complex ways. Just as the association between, for example, an apple and an apple tree is dependent on a higher level concept of seasons and weather, so the challenge faced by the brain is to represent associations in ways that are sensitive to context and to more remote second- and third-order associations. And within this framework, not only does the model become complex and intricate, but the circumstances under which it must be updated become ever more opaque. Consider seeing an apple tree with no apples: there are numerous reasons why this PE should not be a reason for updating our association between apples and apple trees, and the likelihood of modifying our belief about this association would depend on many factors. Computational models coming to grips with this complexity are producing insights that show great promise for informing and testing hypotheses about mechanisms by which psychosis – aberrant world modelling – may emerge (Adams et al., 2013; Corlett and Fletcher, 2014; Fletcher and Frith, 2009).

Thus, there were a number of important parameters that were not part of our model, and we consider these in the latter part of this article, showing how their inclusion enriches, without substantially changing, the basic ideas adumbrated in the original article. In particular, we consider how an aberrant PE signal, over time, could lead to adjustments in attention and perception, as well as the readiness to update one’s model of the world in response to new information (Adams et al., 2013; Corlett and Fletcher, 2014; Fletcher and Frith, 2009). In this richer and more nuanced consideration, we find promising ways of extending the model to explain other key aspects of delusions, as well as important accompanying features such as hallucinations and negative symptoms (Adams et al., 2013; Corlett, 2015; Corlett and Fletcher, 2014; Fletcher and Frith, 2009).

Prior to this, we focus on how the basic theory, as originally outlined, has fared empirically and how it relates to other accounts of delusions (Coltheart and Davies, 2000) and to schizophrenia more broadly.

## Empirical approaches to the PE model of delusions

In 2007, we made the case that ketamine infusion in healthy people provided a window on a hitherto experimentally challenging situation: the emergence of psychotic experience and belief. With the advent of early intervention approaches in psychosis and studies of the psychosis prodrome, it became possible to study patients closer to this illness phase. Furthermore, the continuum from healthy beliefs through to delusions has been increasingly appreciated. Studying attenuated psychotic symptoms (in otherwise healthy subjects) has proven a fruitful avenue of inquiry for testing the model. It should be noted that the basic experimental design that we have initially favoured, in provoking PE signal in order to characterise neural responses using functional neuroimaging, has been challenged (Griffiths et al., 2014). Having responded to this challenge (Corlett and Fletcher, 2015), we do not propose to revisit the argument here, but do note that the fundamental ideas underlying the model were applauded and the overall evidence in their favour was considered robust (Griffiths et al., 2014). There is a more fundamental challenge relating to whether a PE signal disruption is a sufficient circumstance to engender delusions, and given that this relates to a long-standing dialogue among delusion theorists, we consider this in more detail below.

Before considering the challenges to the model and its shortcomings, it is reassuring to note that there is a good deal of support for the idea that psychosis is associated with altered PE signalling. Importantly, in a study of patients with first-episode psychosis, using an identical task, we observed a very similar pattern of PE responses in the right dorsolateral prefrontal cortex as that seen in healthy people administered ketamine (Corlett et al., 2006, 2007b). Crucially, these aberrant responses correlated with the severity of altered beliefs across subjects. In the same participants, using a different (reward-based) learning task, we observed a pattern of altered PE response that was highly comparable to controls (Murray et al., 2008). In both tasks, causal learning and reward PE signals in frontal, striatal and midbrain regions were inappropriately engaged. However, only rDLPFC PE during causal belief formation correlated with delusion scores in patients with first-episode psychosis. In healthy, non-psychotic people too, the degree to which their prefrontal PE response resembles that observed in patients with delusions correlates with the distress they feel with regard to their delusion-like ideas, though ventral striatal responses associated with the ideas themselves, irrespective of accompanying distress (Corlett and Fletcher, 2012). That is, if you hold your beliefs like a patient with psychosis, your right frontal PE response approximates that observed in patients with delusions (Corlett and Fletcher, 2012). These data came from our own work. Others have similarly observed aberrant PE signals in striatum, amygdala and frontal cortex in patients with psychotic illness that correlate with the severity of delusions (Gradin et al., 2011; Romaniuk et al., 2010; Schlagenhauf et al., 2009; Waltz et al., 2010).

In short, empirical data from patients with psychosis have consistently linked aberrant PE signal (measured with functional magnetic resonance imaging in various task contexts; electroencephalogram [EEG] and magnetoencephalogram correlates of PE have yet to be related to delusions) to the severity of delusions. However, there have been a number of theoretical and empirical challenges to the model that we now go on to discuss.

## Shortcomings of the 2007 model

When we presented the model in 2007, it was an initial sketch of how ketamine might give rise to delusion-like ideas. While it gave us some elbow room to begin carving a more complete explanation of clinical delusions in terms of mind and brain function, it was by no means complete. It did not, for example, address one of the cardinal features of delusions: their fixity in the face of contradictory evidence. Indeed, it could be argued that in positing a model that could explain how beliefs are too readily updated, we were inherently failing to explain why the new beliefs themselves – the delusions – are actually tenacious and seemingly immune to new and contradictory evidence.

Moreover, the model had little to say directly about the content of delusions and in particular their focus on the social realm (the fact that they are often about other people and one’s relationships to them). Nor did it deal with the affective charge of delusions. After all, why are delusional beliefs so deeply coloured by emotion and mood? Finally, delusions do not occur in isolation. They are frequently accompanied by hallucinations and often co-occur with negative symptoms such as social withdrawal, apathy and self-neglect. We consider each of these in turn.

The main advance since 2007 involves an appreciation that models of associative learning might pertain not just to animal conditioning and human beliefs but also to perception. This allows us to address many of the earlier model’s shortcomings. It entails conceiving of perception not as a passive process of sensory reception, but rather as active analysis by synthesis.

Our perceptual experience comprises not the actual sensory input but rather the most likely (based on prior experience) cause of this input. We do so by exploiting past regularities (encapsulated in our world model). Herman Von Helmholtz called this ‘unconscious inference’ and made the provocative claim that all perception was a form of controlled hallucination (given how reliant perception is upon these prior regularities rather than raw sense data). Pavlov also appreciated the deep connection between conditioning and perception: ‘Evidently what the genius Helmholtz was referring to in unconscious inference, is the mechanism of the conditioned reflex’ (Pavlov, 1928).

At the neuro-computational level, these ideas are realised in the hierarchical organisation of the brain. Prior expectations are realised top-down via NMDA and GABA signalling. Any mismatch between those priors and incoming information is signalled bottom-up via AMPA receptors. The impact of a given PE on future predictions is governed by its precision (or inverse variance). This computational motif is recapitulated across successive layers in a hierarchical manner – moving away from raw sensory data, the representations become increasingly complex, multifaceted (Mesulam, 1998) and perhaps distant from the immediate evidence of perception. The priors from the level above impact the signals from the level below. Different neuromodulators (dopamine, acetylcholine, serotonin, oxytocin) may implement the precision of priors and PEs in particular processing hierarchies.

## Is altered PE signalling enough to explain delusions?

The PE account of delusions can be considered a one-factor account in that it argues that disruption of a single process (or collection of interrelated processes) may suffice to explain such beliefs. This raises a conflict with neuropsychological models asserting that two factors are necessary for delusions to form (a perceptual disruption and a belief evaluation deficit). We argue that actually this conflict is more apparent than real and arises because the PE account is pitched at a different level of explanation to that of such neuropsychological accounts.

The argument for there being two necessary factors (disruptions) in delusions is compelling. It is often related to monothematic delusions following brain damage, although it has been applied to patients with schizophrenia who have delusions. The main focus of the two-factor theory is Capgras delusion – the belief that a loved one has been replaced by an imposter – and it has been suggested that it can only arise when two things happen. First, a person fails to show the normal autonomic response to a person (leading to a lack of feeling of familiarity, even though there is a strong recognition). This would fit with the unlikely explanation that this person has been replaced, but such an explanation would only be accepted (i.e. the delusion would only form) if the sufferer also had a deficit in their ability to evaluate and reject improbable beliefs. An advantage of this explanation is that it is based on standard neuropsychological methodology and draws on the existence of a single dissociation across the two factors (the experience of a lack of sense of familiarity and the emergence of the ensuing belief).

The two-factor theory implies a separation between perception and cognition. The essence of the predictive processing approach that underpins the PE model of delusions is that such separation, though it functions well at a descriptive level (some mental phenomena can meaningfully be described as beliefs and some as perceptions) does not require a separation at a deeper level. More specifically, it argues that both perceptions and beliefs are inferences (based upon an integration of upcoming data and existing prior expectation). Though they act at different levels within the hierarchy, the fundamental processes that underpin them and that may cause their perturbation may be common. In our model, hierarchy is key. Factor one (altered experience) could be specified lower in the hierarchy, and factor two (altered belief evaluation) higher up. But crucially, PE and its resolution unite them. Furthermore, enough disruption low down or high up can result in delusions.

It has been argued that the two-factor theory subsumes a PE model, but that the PE model alone is insufficient, since it only explains the abnormal experience but not the altered belief. We disagree with this perspective. While it is compelling to us that PE may offer a good explanation for abnormal experiences, we encourage the question of what the effects might be of a perturbed PE signal as we move up to higher, more abstract and complex levels of inference. There, we would see a disruption in the ability to evaluate and, where appropriate, reject, models of the world. In short, viewed at this deeper level, the same process that accounts for abnormal perception can also account for abnormal belief.

Simply put, the two explanations (two-factor and predictive processing) are cast at different explanatory levels. The two-factor theory is concerned with describing cognitive architectures. Predictive processing aims to unite brain, behavioural and phenomenological data for all delusions (neurological and those that occur in schizophrenia) and, as we argue presently, other psychotic symptoms such as hallucinations and amotivation.

The psychologist Kurt Lewin coined the aphorism ‘there is nothing as practical as a good theory’. Since 2007, this expanded hierarchical model has been applied to explain other aspects of delusions, other psychotic symptoms (hallucinations and negative symptoms) and the psychotomimetic effects of other interventions (serotonergic hallucinogens, tetra-hyrdo-cannabinol [THC]). We now enumerate some of those advances.

### Why do delusions persist?

One remarkable feature of delusional beliefs is their elasticity: they expand and morph to include new contradictory data. The person with Capgras, claiming their spouse is an imposter, might respond to other family members who greet the spouse warmly by saying, ‘Of course they hug her [the impostor spouse] – they’re in on it!’ This is difficult to understand in at least two respects. First, the sufferer can often learn about other new things (they don’t have an all-encompassing deficit in learning), so why do they seem unable to incorporate new and often strongly contradictory evidence into their beliefs rather than so tenaciously holding the central delusional belief? Second, and related to this, the PE model of delusional emergence hypothesises an inappropriately enhanced tendency to develop new beliefs. Prima facie, this would surely militate against those beliefs being unshakeable.

In trying to answer these questions, we pursued two related lines of thought. The first concerns a disruption in the ways in which newly learned associations become updated. The idea here is that delusions differ from other beliefs in several ways that change their encoding and reconsolidation in memory. First, we suggest, their emergence occurs in response to a world that has become strange and mysterious. There is a puzzle to be solved. Compelling coincidences and seemingly significant events provoke a search for meaning, and the belief that eventually seems to resolve the ambiguity and uncertainty has a powerful function in relieving stress and anxiety. The fact that the belief is often unpleasant does not detract from this function, since it may be easier to bear and respond to a difficult certainty than a nameless and shapeless fear. Given their explanatory utility, they are rehearsed extensively. When delusions are questioned, bringing them to mind may actually serve to reinforce rather than to disrupt the memory (Corlett et al., 2010; Corlett et al., 2009b; Corlett et al., 2013). The idea here is that re-evocation of an association may, under certain circumstances (notably when PE signalling is inherently disrupted), strengthen a memory, even when it is not formally reinforced. We have modelled this process in humans with ketamine. By creating new associations (either appetitive or aversive) and then, a day after, reactivating them (in the absence of the original reinforcer) under ketamine, we observed that the memories became strengthened in comparison to the same procedure under placebo (Corlett et al., 2013). Indeed, the magnitude of this effect correlated with ketamine-induced psychosis and PE brain signal (Corlett et al., 2013). We replicated this memory-enhancing effect in rodents (Honsberger et al., 2015). Conditioned fear memories reactivated under ketamine are subsequently strengthened (Honsberger et al., 2015). This effect was blocked by ifenprodil infusion in the amygdala (a procedure commonly used to block memory destabilisation and updating in preclinical studies of reconsolidation; Honsberger et al., 2015).

The above, empirically supported but currently tentative explanation for how a belief begins to strengthen, even in the absence of objective evidence, can be considered alongside another important phenomenon relating to PE signal. Specifically, there is growing evidence that we are sensitive not just to the magnitude of PE but also to its variability. A high degree of variability can be encoded and leads in time to an adaptation of learning such that a given magnitude of a specific PE produces less updating (Diederen and Schultz, 2015; Preuschoff and Bossaerts, 2007; Preuschoff et al., 2008). In effect, we encode not just surprise or the unpredictability of a single event, but also keep a running tally of how likely we are to be able to predict the current environment, downregulating PE-dependent learning when our best predictions are unable to reduce PE. Thus, one can envisage that in the emergence of psychosis, a person is not just updating beliefs to suppress PE, but also, at a larger timescale, beginning to downregulate the importance of PE. This could lead to the possibility that early beliefs persist and can then remain relatively unchallenged as the person adapts learning rate (learning not to update). Again, this is speculative, but it perhaps demonstrates the enhanced power to the PE model of taking into account dynamic, evolving effects and, importantly, suggests further experiments.

### What may account for the characteristic contents of delusions?

Our original article was primarily concerned with describing a mechanism by which delusions might arise and had little to say about the characteristic content of delusional beliefs. Here, we outline some ideas that may fill this gap in relation to four characteristics of delusional thinking. First, delusions are highly personal in one sense, reflecting the fears and preoccupations of the individual, while at the same time, there are more generic and common aspects to them, and they draw more broadly on the contents of that individual’s culture and era (Stompe et al., 2003). Second, delusions usually relate to agents and can often seem to reflect a fundamental alteration in the ability to attribute agency appropriately. Third, related to this, delusions are predominantly social (Bell, 2013; Fineberg and Corlett, 2016). They are often about people and their intentions or goals rather than merely physical entities. Thus, for example, a person may come to view an array of unusual coincidences and sinister experiences as arising from the actions of a persecutor (Kihlstrom and Hoyt, 1988). We note of course that there are some delusions with apparent positive content, and have recently argued that delusions may serve an adaptive function of facilitating continued engagement with an unpredictable environment (Fineberg and Corlett, 2016). Ultimately though, even grandiose delusions can be a source of distress and uncertainty (the person may feel themselves to be responsible for important events, including unpleasant ones, and may see themselves as vulnerable to envious and powerful enemies; Corlett et al., 2007a).

The first characteristic is, in one sense, very straightforward to understand. The delusion is seen as a person’s hypothesis about the origins of their perceptual experiences. It is, as Coltheart et al. (2010) have observed, an abductive inference in which data are used to infer their underlying cause. Given that such inference relies on a person’s best guess (Pierce, 1931–1958), then it follows that their own prior knowledge and expectation will necessarily determine the content of the emergent belief. And since their own expectations are, inter alia, socioculturally determined, there will be a strong overlap between those of the person and the time and culture they inhabit. The shifting nature of delusions across the decades testifies to this (Stompe et al., 2003).

A further idea that emerges from this model is that if the delusion arises to explain uncertainty, it seems feasible that certain features of our environment, being inherently more uncertain, may prove more likely to become the subject of delusional thoughts. Phenomena such as the intentions of others, their hidden goals, the import of their actions and their facial expressions may all be areas that are most likely to change in the face of altered PE (Corlett, 2015).

The question of agency in delusions, and psychosis more generally, is a very interesting one. While delusions are by no means uniquely about agents, they are nonetheless frequently preoccupied with the intentions and actions of other agents, and, in some cases, may explicitly entail a sense that another agent is exerting control over oneself (Frith, 2005a, 2005b). More broadly, other psychotic symptoms that can co-occur with delusions such as hearing voices have also been attributed to a failure to attribute agency correctly such that one’s own inner speech feels as though it has been externally generated (Ford, 2016; Ford and Mathalon, 2005; Ford et al., 2007). This has been framed in terms of a general source-monitoring deficit in schizophrenia (Keefe et al., 1999; Keefe and Kraus, 2009; Kraus et al., 2009).

In fact, there is a compelling body of theoretical literature relating prediction and expectation to the attribution and experience of agency. This comes from observations that even under normal circumstances, it is possible to produce a sense of agency in people who are not in fact the authors of an action and, conversely, to disavow agency for their own actions (Frith, 2005a, 2005b). Both of these phenomena can be produced by altering expectations and external cues, leading to the emerging view that sense of agency emerges from the integration of prior expectations with internal (e.g. proprioceptive) and external cues (Moore et al., 2011a; Moore and Fletcher, 2012). This has been very well-illustrated in the work of Daniel Wegner who shows that both expectations as well as external cues can profoundly alter the degree to which one sees oneself as the cause of events or attributes them to some external agent (Wegner and Wheatley, 1999).

In this sense, attribution of agency has the same status as other abductive inferences characterising delusional thinking (as discussed above), and perturbations within the system, as would be the case with disrupted PE signalling, could fundamentally alter the experience both of one’s own agency and of that attributed to events in the outside world. Put in simple terms, one’s own actions are characterised partly by the predictability of their consequences, so an action accompanied by an unpredictable consequence is perhaps more likely to originate externally (Frith, 2005a, 2005b).

There is some evidence for the above perspective. Ketamine also enhances intentional binding (the perceived compression in time between action and outcome for deeds for which we feel agency; Moore et al., 2011b). Intentional binding is likewise enhanced in patients with first-episode psychosis (Hauser et al., 2011; Voss et al., 2010). PEs have been implicated in intentional binding effects; binding effects are subject to Kamin blocking (prior learning of action–event associations can block the intentional binding effect). The blocking is weaker in individuals with higher schizotypy scores (Moore et al., 2011a). Furthermore, one’s sense of agency for actions can be probed by considering forward modelling comparing predictions to feedback sensations and cancelling what was predicted to guide one’s sense of ownership for thoughts and actions. Specifically, we are more likely to feel ourselves to be agents when the consequences of actions are predicted and more likely to assume external agency when they are unpredicted. It has been shown that for self-produced forces, we are likely to cancel out the sensory consequences (which are predicted) such that when trying to match an external force that we have just experienced on our finger by pressing down on the same finger, we overcompensate. The extra force exerted is thought to overcome the cancellation effect of our anticipation prior to performing an action (Shergill et al., 2003, 2005).

Patients with schizophrenia do not overcompensate; they are more accurate (Shergill et al., 2005). Also, more accurate force matching (without predictive over-compensation) correlates with delusion-like ideation in healthy people (Teufel et al., 2010). Taken together, these data confirm that PE-driven inferences are central to a range of experiences. Ketamine and psychosis similarly perturb these PEs, and those perturbations relate to the severity of endogenous and ketamine induced psychotic symptoms (Moore et al., 2011b).

PEs have been invoked to explain the sense of agency for our actions and ownership for our bodies. Ketamine augments experience of the rubber-hand illusion – the spurious sense of ownership of a prop-hand if the hand is stroked at the same time as one’s own hand (Morgan et al., 2011). People on ketamine get the illusion more strongly, and they experience it even in a control condition when the real and rubber hands are stroked asynchronously (Morgan et al., 2011). Patients with schizophrenia (Peled et al., 2003) and chronic ketamine abusers evince the same excessive experience of the illusion in the synchronous and asynchronous conditions (Tang et al., 2015). Activity in the right anterior insula cortex increases to the extent that individuals experience the illusion. Anil Seth and others have argued that the anterior insula is a key nexus for the PE-driven inferences that guide perceptions of bodily ownership and agency (Palmer et al., 2015; Seth, 2013; Seth et al., 2011).

In the remainder of the paper, we attempt to bring other symptoms – specifically, hallucinations and negative symptoms – into the explanatory fold. In so doing, we consider new dimensions of the theory (including its relationship to artificial intelligence and deep learning).

## Predictive processing and hallucinations

Can predictive processing theory explain hallucinations? These have been related to aberrant PE signals in primary sensory cortices (Horga et al., 2014). Furthermore, prior theories of hallucinations can be cast in predictive processing terms. For example, auditory verbal hallucinations (AVH; ‘voices’) have been explained as aberrations of predictive forward models of inner speech (Ford, 2016; Ford and Mathalon, 2005; Ford et al., 2007). This corollary discharge explanation posits that thoughts and inner speech are prosecuted by means of an efferent copy of the motor acts that such inner speech would entail. This efferent copy from cerebellum to parietal cortex is used to cancel the sensorimotor consequences’ actions (Blakemore et al., 1999). This mechanism may explain delusions of motor control in which a person experiences their own actions as arising from, and being controlled by, an external agent.

The same theory has been applied to inner speech (Feinberg, 1978) – that we predict the consequences of speaking in our heads (Frith, 2005a, 2005b). Any mismatch leads to the perception of alien agency for the inner speech – it is perceived as external. Accordingly, having subjects open their mouth wide, preventing pre-articulatory motions of the facial muscles, may well attenuate AVHs, perhaps because of effects on these motor predictions (Bick and Kinsbourne, 1987).

Wilkinson (2014) recently cast this model in predictive processing terms. The phenomenology of AVH comes about because the individual infers the best explanation for unexpected inner speech that reaches the threshold for awareness must be an individual talking inside one’s head. Despite its intuitive appeal, corollary discharge or efferent copy theory has not fared so well empirically (Ford, 2016). Corollary discharge processing as measured by frontal cortex EEG signals during speech production and the perception of perturbed versions of one’s own speech is impaired in patients who hear voices, but it is likewise impaired in patients with schizophrenia who do not have AVH (Ford, 2016). Furthermore, the severity of corollary discharge impairments does not correlate with AVH severity across subjects (Ford, 2016). Finally, recent work suggests that rather than a failure of prediction that subtends aberrant PE, hallucinations may come about via an undue influence of priors on current processing (Teufel et al., 2015). Patients with an at-risk mental state are more likely to use prior visual information in making visual decisions (Teufel et al., 2015).

Taken together with observations of conditioned hallucinations (sensory experiences without stimuli that can be trained in the lab and to which patients with AVH are more sensitive; Kot and Serper, 2002), the empirical data suggest that hallucinations and delusions, whilst related, may be differently driven by the specification of prior expectations and how they shape subsequent processing. How can this be? In what follows, we focus on elaborations of the PE model that might explain the genesis of hallucinations, as well as the co-occurrence of positive and negative symptoms.

## Reliability and uncertainty

To explain how hallucinations, delusions and perhaps even negative symptoms can co-occur despite being related to subtly different aspects of predictive processing, we turn to statistical learning theory, in particular how learning theories deal with attentional allocation. Many different, sometimes opposing, events can be salient. Importantly, both unpredicted events and those that are consistent predictors of important outcomes are potentially salient. These types of events (predictive and unpredicted) seem to have very different relationships to prediction. Statistical models of attentional allocation, unlike many formal learning theory models, allow for this. They simultaneously assess the relevance of stimuli for predicting outcomes (this is considered as their ‘reliability’), and the relevance of their uncertainty, or failure to predict outcomes correctly for adjusting the predictions (Dayan et al., 2000). Considering the above, simple example of the apple tree, this might be a highly reliable predictor of the presence of apples, given that it does much better than other trees, but nonetheless a very uncertain one (given the importance of the seasons or weather).

Reliabilities lead to competition between stimuli for making the predictions (Mackintosh, 1975) and so are different from uncertainties in prediction which lead to competition for learning the predictions (Pearce and Hall, 1980). Reliabilities are the statistical account of which stimuli we deem important predictors, whereas uncertainties quantify how well those predictions are known. Reliability and uncertainty have different neurobiological and neurochemical mechanisms (Yu and Dayan, 2005). They have opposing relationships to PE. We argue that ketamine-induced positive and negative symptoms may relate to impaired reliability and enhanced uncertainty processing, respectively.

Both reliability and uncertainty affect attentional allocation. Organisms attend to reliable predictors of salient events (Anderson et al., 2011; Mackintosh, 1975), but on the other hand, PEs elicit surprise. Therefore, reliable events amass fewer PEs and should therefore be attended to less than unreliable predictors (Pearce and Hall, 1980). How can this be? Holland and Schiffino (2016) propose that the different ecological demands on attention in learning and action selection may explain the difference. Action decisions are optimised through a bias towards attending to reliable predictors of future states. On the other hand, learning is best served by focusing on the unknown. Pearce and Hall (1980) made this distinction by contrasting controlled versus automatic processing. The learning rate or associability of cues may not be equivalent to that for actions. Attention in learning may be driven by PEs. For action, predictions may dominate. For example, animals can attend to one element of a stimulus array for action guidance (based on its reliability) and another independent feature for learning (based on uncertainty). In the five-choice serial reaction time task, rats can be cued to which action to select. Degradation of those cues (by shortening the cues or decreasing their salience) can increase both errors of commission and omission. The more reliable predictors of which action to commit garner the most attention. On the other hand, those same cues can be learned as probabilistic predictors of food outcomes. Here, uncertain Pavlovian predictors accrue attention and are thus subsequently more associable: they are more readily learned about. In rodents, lesions of medial prefrontal cortex and parietal cortex doubly dissociate reliability-based from uncertainty-based attention (Holland and Schiffino, 2016).

We believe that this distinction may be helpful in reconciling the co-occurrence of delusions, hallucinations and negative symptoms in the same patients. These disparate (seemingly contradictory) symptoms may be differentially reliant on impairments in reliability processing (action selection) or uncertainty processing (learning).

Given the association between negative symptoms and goal-directed action selection, we might predict that patients with schizophrenia might show attenuated responses to cues that guide action selection (impaired reliability estimates). At the same time, patients with delusions might show spurious responses at the time of the outcome (enhanced uncertainty). Data from the monetary incentive delay task support this assertion. Negative symptoms in people with schizophrenia correlate with attenuated striatal responses to action eliciting cues that portend, for example, which action to select and its associated value (Waltz et al., 2010). On the other hand, positive symptoms (specifically delusions) correlate with aberrant PE signals in lateral prefrontal cortex (Corlett et al., 2007b; Waltz et al., 2010), medial prefrontal cortex (Schlagenhauf et al., 2009) and midbrain (Romaniuk et al., 2010) at the time of the outcome.

Predictive processing theory is cast in terms of the hierarchical arrangement of neural systems. Priors are specified top-down and PEs communicated bottom-up, but at each hierarchical level, there may be different relative precisions of predictions and PE (reliability and uncertainty, respectively). And these trade-offs may be different for visual, auditory, motor and other hierarchies. Thus, it may be that a global PE dysfunction (e.g. from disrupted excitatory inhibitory balance in the cortex; Bastos et al., 2012) may impact these hierarchies to different degrees, and to the extent that specific hierarchies (perceptual, motor) are disrupted, different symptoms (positive and negative) might arise.

A brain that is receiving noisy signals can become hungry for the priors that could possibly make sense of that noise and thus resolve uncertainty (Teufel et al., 2015). Thus, when signal from the lowest levels of sensory input are noisy, it may impose precise priors top-down higher in the hierarchy – weighting perception towards expectation (rather than input) in a listening attitude as Arieti (1974) put it – which produces hallucinations (Hoffman, 2010). Sensory isolation can engender the same effect as bias towards prior expectations that engenders hallucinations (Corlett et al., 2009a).

Overwhelming unreliability of previous actions will likely produce a change in one’s higher-level beliefs about the efficacy of one’s action, perhaps leading to the conclusion that no behaviours will be effective at reducing uncertainty, so it is best not to act at all, producing negative symptoms (Corlett, 2015).

## Other drug models

In 2007, we focused on predictive learning and ketamine. By expanding the model in terms of predictive processing and its role in perception and action, we were able to bring the psychotomimetic effects of other drugs into the explanatory fold. Serotonergic hallucinogens such as LSD induce visual hallucinations (Geyer and Vollenweider, 2008).

They do not, however, induce delusions (Young, 1974). In rats, they enhance glutamatergic responses to sensory stimuli in the locus coeruleus (Rasmussen and Aghajanian, 1986) and frontal cortex (Aghajanian and Marek, 1997), and may actually enhance NMDA signalling (Lambe and Aghajanian, 2006). Excessive AMPA signalling in the absence of NMDA impairment would lead to increased sensory noise in the context of normal priors. This is exactly the context in which we expect hallucinations to arise. The neural correlates of priors and PEs, top-down and bottom-up, have yet to be completely delineated. One theory of the default mode network, a brain circuit engaged when subjects are in a task-free mind-wandering state, is that it reflects PEs to be explained and the process of learned resolutions (Carhart-Harris and Friston, 2010). Rodent data support this idea (Berkes et al., 2011). Serotonergic hallucinogens increase default mode responses in human subjects in a manner that correlates with their psychotomimetic effects (Carhart-Harris et al., 2013). However, behavioural tasks that engage associative learning, perception and belief formation have yet to be examined in this context.

Cannabinoids such as Δ-9-THC also have psychotomimetic effects (D’Souza et al., 2004). The binocular depth inversion illusion (a stereoscopic effect thought to be driven by prior expectations about stimulus curvature) is attenuated by Δ-9-THC (Koethe et al., 2006; Semple et al., 2003). This weakening of top-down influences would suggest that hallucinations should not predominate under Δ9-THC administration, and this appears to be the case. However, delusion-like ideas do occur (D’Souza et al., 2004).

Amphetamine elevates dopamine levels in the striatum in healthy volunteers and more so in individuals with schizophrenia (Laruelle et al., 2003). A single dose of amphetamine does not induce delusion-like ideas or hallucinations. Rather, elevated mood, grandiose ideas and hyperactivity are more characteristic (Jacobs and Silverstone, 1986). It also increases perceptual acuity of the whole visual field (Fillmore et al., 2005), unlike ketamine, which enhances the salience of discrete and apparently random objects, events and stimuli (Corlett et al., 2007a; Oye et al., 1992). We suggest that this pattern of psychopathology is due to increased precision of both priors and PEs through enhanced dopaminergic (Kegeles et al., 1999; Laruelle et al., 1995) and cholinergic function (Acquas and Fibiger, 1998).

In sum, considering the trade-off between prior experiences and current inputs via their relative precision opens a whole new explanatory scope for the model, both in terms of symptoms other than delusions and interventions other than ketamine (Corlett et al., 2009a).

## The future: The emergence of computational psychiatry

It would be remiss not to acknowledge the fact that the PE model of delusions was formulated in the setting of exciting developments in the application of computational models to psychiatric questions, and arrogant to fail to acknowledge that computational psychiatry, though it has come into the spotlight fairly recently (Corlett and Fletcher, 2014; Friston et al., 2014; Montague et al., 2012), has a long history.

Some of the earliest work in artificial intelligence (AI) was rapidly used to explore the genesis of psychosis. For example, symbolic models of natural language were trained to implement simple responses to verbal questions. By altering the model parameters (e.g. its input–output mappings), paranoid responses could be elicited (Colby, 1960). Hoffman and Dobscha (1989) went beyond the language of thought analogy, implementing an artificial neural network model called a Hopfield network that could memorise inputs and give appropriate outputs. By pruning the allowable connections in this model, it produced spurious recall that Hoffman and Dobscha (1989) related to hallucinations and delusions. Before his untimely death, Ralph Hoffman combined Hopfield networks with a modular cognitive architecture to examine story learning and recall. When he increased PE signalling in the network, it began recalling spurious agents in the narratives it produced, inserting itself into those stories in a manner consistent with some delusions (Hoffman et al., 2011). Again, these networks were not hierarchical.

We and others have argued that hierarchical organisation is likely a key organisational principle for cortical processing. This principle is likewise captured in ‘deep learning’ (LeCun et al., 2015) – state of the art AI that involves hierarchical (or ‘deep’) architectures comprised of many Hopfield networks, separated by hidden layers, that learn representations of data without supervision and use reinforcement learning on these representations to guide action selection, beating humans at Atari games (Mnih et al., 2015) and Go (Silver et al., 2016).

In deep learning, each stage in the hierarchy learns to generate or reconstruct the activation patterns in the stage below. One such network, the Deep Boltzmann machine (DBM), utilises both feedforward and feedback processing (Salakhutdinov and Hinton, 2012), which better suits the recurrent processing in brain hierarchies we have described. In a DBM, each hidden layer receives input from a layer below (that conveys bottom-up information), and from a layer above that has learned predict the activity of the layer below. The hidden units learn latent variable representations of the input data. This means a deep network can synthesise representations of input data, even in the absence of such data (Yuille and Kersten, 2006).

Such network behaviour suggests these architectures might implement Helmholtz’s analysis by synthesis and may be particularly helpful in examining the genesis of hallucinations. Indeed, Reichert et al. (2013) have done just that, using a DBM to model the genesis of visual hallucinations in Charles Bonnet Syndrome, a syndrome occurring in association with visual deficits (e.g. macular degeneration) in which illusory percepts and sometime complex hallucinations occur. As in Yu and Dayan (2002), acetylcholine can be used as a model parameter to set the balance in between feedforward and feedback flow of information in perception. In a version perhaps most relevant to our concerns, Dayan and Hinton describe a Helmholtz machine that uses acetylcholine to trade off priors and PEs and minimise free energy (Dayan et al., 1995; Hinton and Dayan, 1996). This formulation has much in common with the Kalman Filter approach to predictive learning that can embody statistical reliabilities and uncertainty and employs noradrenaline and acetylcholine to do as such (Dayan et al., 2000). These neurotransmitters may be opponent (Yu and Dayan, 2005) and have recently been implicated in the genesis of hallucinations (Collerton et al., 2005; Geddes et al., 2016). Kersten et al. (2004) further characterise a generative model as ‘strong’ if samples can be produced from it that consistently look like the data it learned from (Kersten et al., 2004). Reichert et al. (2013) were able to read out which particular stimuli were being hallucinated, which is a significant advance on earlier work with Hopfield networks.

## Summary and conclusion

In conclusion, our 2007 model offered a rudimentary framework for thinking about how delusions may emerge, one that linked the experiences, via a cognitive model of associative learning, to neural processes. Here, we have shown how an extension of the model – one that brings in related parameters (precision, reliability, certainty) and a more dynamic view of how PE learning might change as PE signal evolves – offers new breadths of explanation. A key next step is to turn some of these insights into practical benefits for the patients who, along with their families and friends, may suffer greatly with their experiences. We believe that the elucidation of mechanisms by which these experiences arise is a necessary prelude to a comprehensive and precise diagnostic system, as well as to the development of individually targeted interventions.
